# 2-Chloro-*N*′-(2-chloro­benzyl­idene)benzohydrazide

**DOI:** 10.1107/S1600536809043803

**Published:** 2009-10-28

**Authors:** Dong-Hui Zou, Hong Guan, Xiao-Hua Zhang

**Affiliations:** a College of Life Science and Engineering, Qiqihar University, Qiqihar 161006, People’s Republic of China; b Qiqihar Medical University, Qiqihar 161006, People’s Republic of China; c Liaoning Cheng Da Biotechnology Co Ltd, Shenyang 100044, People’s Republic of China

## Abstract

The mol­ecule of the title compound, C_14_H_10_Cl_2_N_2_O, adopts an *E* configuration about the C=N bond. The dihedral angle between the two benzene rings is 79.7 (2)°. In the crystal structure, mol­ecules are linked by inter­molecular N—H⋯O, C—H⋯Cl and C—H⋯O hydrogen bonds, forming chains running along the *b* axis.

## Related literature

For the biological activity of hydra­zones, see: Küçükgüzel *et al.* (2003 ▶); Charkoudian *et al.* (2007 ▶); Avaji *et al.* (2009 ▶); Kümmerle *et al.* (2009 ▶); Raparti *et al.* (2009 ▶); Bayrak *et al.* (2009 ▶); Hearn *et al.* (2009 ▶). For crystal structures of hydrazone compounds, see: Fun *et al.* (2008 ▶); Lo & Ng (2009 ▶); Ren (2009 ▶); Zhang (2009 ▶); Wu (2009 ▶); Peng & Hou (2008 ▶); Mohd Lair *et al.* (2009 ▶); Liang & Zou (2009 ▶).
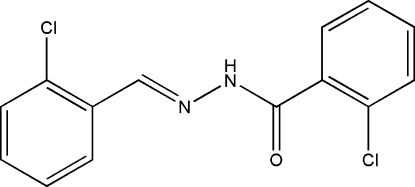


## Experimental

### 

#### Crystal data

C_14_H_10_Cl_2_N_2_O
*M* *_r_* = 293.14
Orthorhombic, 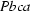

*a* = 11.9336 (5) Å
*b* = 9.7471 (4) Å
*c* = 22.5840 (9) Å
*V* = 2626.93 (19) Å^3^
*Z* = 8
Mo *K*α radiation
μ = 0.49 mm^−1^
*T* = 298 K
0.23 × 0.21 × 0.20 mm

#### Data collection

Bruker SMART CCD area-detector diffractometer
Absorption correction: multi-scan (*SADABS*; Sheldrick, 1996 ▶) *T*_min_ = 0.897, *T*_max_ = 0.909
15167 measured reflections
2863 independent reflections
2035 reflections with *I* > 2σ(*I*)
*R*_int_ = 0.045

#### Refinement

*R*[*F* ^2^ > 2σ(*F* ^2^)] = 0.038
*wR*(*F* ^2^) = 0.098
*S* = 1.03
2863 reflections
175 parameters
1 restraint
H atoms treated by a mixture of independent and constrained refinement
Δρ_max_ = 0.21 e Å^−3^
Δρ_min_ = −0.36 e Å^−3^

### 

Data collection: *SMART* (Bruker, 1998 ▶); cell refinement: *SAINT* (Bruker, 1998 ▶); data reduction: *SAINT*; program(s) used to solve structure: *SHELXS97* (Sheldrick, 2008 ▶); program(s) used to refine structure: *SHELXL97* (Sheldrick, 2008 ▶); molecular graphics: *SHELXTL* (Sheldrick, 2008 ▶); software used to prepare material for publication: *SHELXTL*.

## Supplementary Material

Crystal structure: contains datablocks global, I. DOI: 10.1107/S1600536809043803/ci2943sup1.cif

Structure factors: contains datablocks I. DOI: 10.1107/S1600536809043803/ci2943Isup2.hkl

Additional supplementary materials:  crystallographic information; 3D view; checkCIF report

## Figures and Tables

**Table 1:** Hydrogen-bond geometry (Å, °)

*D*—H⋯*A*	*D*—H	H⋯*A*	*D*⋯*A*	*D*—H⋯*A*
N2—H2⋯O1^i^	0.91 (1)	1.920 (12)	2.809 (2)	166 (2)
C7—H7⋯O1^i^	0.93	2.53	3.301 (2)	141
C14—H14⋯Cl2^i^	0.93	2.75	3.620 (2)	156

Symmetry code: (i) 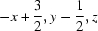.

**Fig. 1.**
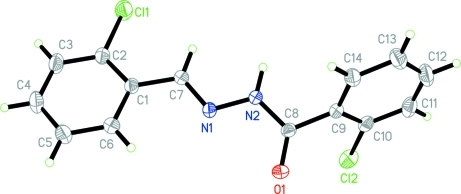
The molecular structure of the title compound, showing 30% probability displacement ellipsoids for the non-hydrogen atoms. H atoms are shown as spheres of arbitrary radius.

**Fig. 2.**
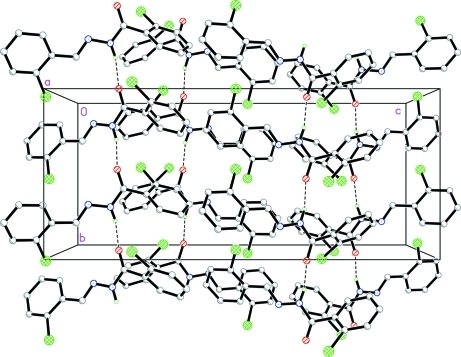
The packing diagram, viewed along the a axis. Hydrogen bonds are shown as dashed lines.

## supplementary crystallographic information

### Comment

During the past decades, the human population affected with life-treating infectious diseases caused by multidrug-resistant Gram-positive and Gram-negative pathogen bacteria increased to an alarming level around the world. Recently, a great deal of antibacterial agents were used in therapy. Hydrazones are an important component of the Schiff base family. These compounds have been widely used in the fields of antimicrobial, antibacterial and antitumor (Küçükgüzel *et al.*, 2003; Charkoudian *et al.*, 2007; Avaji *et al.*, 2009; Kümmerle *et al.*, 2009; Raparti *et al.*, 2009; Bayrak *et al.*, 2009; Hearn *et al.*, 2009). In the last few years, crystal structures of a number of hydrazone compounds have been reported (Fun *et al.*, 2008; Lo & Ng, 2009; Ren, 2009; Zhang, 2009). As a continuation of our work in this area (Liang & Zou, 2009), the author reports herein the crystal structure of the title new hydrazone compound.

In the title molecule (Fig. 1), the dihedral angle between the two benzene rings is 79.7 (2)°. The molecule exists in an *E* configuration about the C═N bond. All bond lengths are within normal values and comparable to those obserbved in related hydrazone compounds (Wu, 2009; Peng & Hou, 2008; Mohd Lair *et al.*, 2009).

In the crystal structure of the title compound, molecules are linked through intermolecular N—H···O hydrogen bonds (Table 1), forming chains running along the *b* axis (Fig. 2).

### Experimental

Equimolar quantities (1.0 mmol each) of 2-chlorobenzaldehyde and 2-chlorobenzohydrazide were mixed and refluxed in methanol. The reaction mixture was cooled to room temperature to give a clear colourless solution. Colourless single crystals of the title compound were formed by slow evaporation of the solution in air.

### Refinement

Atom H2 was located in a difference map and refined isotropically, with the N-H distance restrained to 0.90 (1) Å and U_iso_ set at 0.08 Å^2^. Other H atoms were placed in calculated positions (C-H = 0.93 Å) and refined as riding with *U*_iso_(H) = 1.2*U*_eq_(C).

### Crystal data

**Table:**

C_14_H_10_Cl_2_N_2_O	*F*(000) = 1200
*M**_r_* = 293.14	*D*_x_ = 1.482 Mg m^−^^3^
Orthorhombic, *P**b**c**a*	Mo *K*α radiation, λ = 0.71073 Å
Hall symbol: -P 2ac 2ab	Cell parameters from 2480 reflections
*a* = 11.9336 (5) Å	θ = 2.4–24.5°
*b* = 9.7471 (4) Å	µ = 0.49 mm^−^^1^
*c* = 22.5840 (9) Å	*T* = 298 K
*V* = 2626.93 (19) Å^3^	Block, colourless
*Z* = 8	0.23 × 0.21 × 0.20 mm

### Data collection

**Table:**

Bruker SMART CCD area-detector diffractometer	2863 independent reflections
Radiation source: fine-focus sealed tube	2035 reflections with *I* > 2σ(*I*)
graphite	*R*_int_ = 0.045
ω scans	θ_max_ = 27.0°, θ_min_ = 1.8°
Absorption correction: multi-scan (*SADABS*; Sheldrick, 1996)	*h* = −15→11
*T*_min_ = 0.897, *T*_max_ = 0.909	*k* = −12→12
15167 measured reflections	*l* = −28→28

### Refinement

**Table:**

Refinement on *F*^2^	Primary atom site location: structure-invariant direct methods
Least-squares matrix: full	Secondary atom site location: difference Fourier map
*R*[*F*^2^ > 2σ(*F*^2^)] = 0.038	Hydrogen site location: inferred from neighbouring sites
*wR*(*F*^2^) = 0.098	H atoms treated by a mixture of independent and constrained refinement
*S* = 1.03	*w* = 1/[σ^2^(*F*_o_^2^) + (0.038*P*)^2^ + 0.9372*P*] where *P* = (*F*_o_^2^ + 2*F*_c_^2^)/3
2863 reflections	(Δ/σ)_max_ = 0.001
175 parameters	Δρ_max_ = 0.21 e Å^−^^3^
1 restraint	Δρ_min_ = −0.35 e Å^−^^3^

### Special details

**Table:**

Geometry. All e.s.d.'s (except the e.s.d. in the dihedral angle between two l.s. planes) are estimated using the full covariance matrix. The cell e.s.d.'s are taken into account individually in the estimation of e.s.d.'s in distances, angles and torsion angles; correlations between e.s.d.'s in cell parameters are only used when they are defined by crystal symmetry. An approximate (isotropic) treatment of cell e.s.d.'s is used for estimating e.s.d.'s involving l.s. planes.
Refinement. Refinement of *F*^2^ against ALL reflections. The weighted *R*-factor *wR* and goodness of fit *S* are based on *F*^2^, conventional *R*-factors *R* are based on *F*, with *F* set to zero for negative *F*^2^. The threshold expression of *F*^2^ > σ(*F*^2^) is used only for calculating *R*-factors(gt) *etc*. and is not relevant to the choice of reflections for refinement. *R*-factors based on *F*^2^ are statistically about twice as large as those based on *F*, and *R*- factors based on ALL data will be even larger.

### Fractional atomic coordinates and isotropic or equivalent isotropic displacement parameters (Å^2^)

**Table:**

	*x*	*y*	*z*	*U*_iso_*/*U*_eq_	
Cl1	0.79500 (6)	0.47092 (7)	0.48378 (3)	0.0674 (2)	
Cl2	0.53369 (5)	1.04300 (6)	0.72070 (3)	0.05271 (18)	
N1	0.84153 (13)	0.81563 (16)	0.59949 (6)	0.0345 (4)	
N2	0.78786 (14)	0.79951 (16)	0.65338 (7)	0.0341 (4)	
O1	0.78386 (13)	1.02813 (13)	0.67114 (6)	0.0445 (4)	
C1	0.90227 (16)	0.7097 (2)	0.51022 (8)	0.0352 (4)	
C2	0.88637 (17)	0.6053 (2)	0.46878 (9)	0.0413 (5)	
C3	0.9388 (2)	0.6077 (3)	0.41454 (10)	0.0540 (6)	
H3	0.9271	0.5370	0.3876	0.065*	
C4	1.0084 (2)	0.7148 (3)	0.40032 (10)	0.0560 (6)	
H4	1.0434	0.7167	0.3635	0.067*	
C5	1.02694 (18)	0.8197 (2)	0.44010 (9)	0.0494 (6)	
H5	1.0743	0.8920	0.4303	0.059*	
C6	0.97447 (17)	0.8163 (2)	0.49456 (9)	0.0415 (5)	
H6	0.9876	0.8867	0.5215	0.050*	
C7	0.84564 (16)	0.70741 (19)	0.56775 (8)	0.0359 (4)	
H7	0.8126	0.6268	0.5812	0.043*	
C8	0.76315 (16)	0.91055 (19)	0.68656 (8)	0.0327 (4)	
C9	0.71126 (16)	0.87531 (19)	0.74505 (8)	0.0346 (4)	
C10	0.61214 (17)	0.9330 (2)	0.76526 (9)	0.0387 (5)	
C11	0.5702 (2)	0.9017 (3)	0.82073 (10)	0.0520 (6)	
H11	0.5042	0.9422	0.8339	0.062*	
C12	0.6263 (2)	0.8106 (3)	0.85620 (10)	0.0600 (7)	
H12	0.5982	0.7897	0.8935	0.072*	
C13	0.7236 (2)	0.7499 (3)	0.83710 (10)	0.0570 (6)	
H13	0.7606	0.6869	0.8611	0.068*	
C14	0.76608 (19)	0.7831 (2)	0.78197 (9)	0.0462 (5)	
H14	0.8326	0.7430	0.7694	0.055*	
H2	0.768 (2)	0.7142 (14)	0.6655 (11)	0.080*	

### Atomic displacement parameters (Å^2^)

**Table:**

	*U*^11^	*U*^22^	*U*^33^	*U*^12^	*U*^13^	*U*^23^
Cl1	0.0867 (5)	0.0575 (4)	0.0581 (4)	−0.0282 (3)	0.0204 (3)	−0.0183 (3)
Cl2	0.0438 (3)	0.0540 (4)	0.0603 (4)	0.0084 (3)	−0.0088 (3)	−0.0094 (3)
N1	0.0413 (9)	0.0334 (9)	0.0288 (8)	0.0033 (7)	0.0049 (7)	0.0008 (7)
N2	0.0447 (10)	0.0279 (8)	0.0297 (8)	0.0004 (7)	0.0066 (7)	−0.0014 (7)
O1	0.0614 (10)	0.0267 (7)	0.0452 (8)	0.0046 (7)	0.0093 (7)	0.0024 (6)
C1	0.0396 (11)	0.0358 (11)	0.0301 (10)	0.0058 (8)	0.0022 (8)	0.0013 (8)
C2	0.0448 (12)	0.0416 (12)	0.0376 (11)	−0.0017 (9)	0.0059 (9)	−0.0040 (9)
C3	0.0608 (15)	0.0597 (15)	0.0415 (12)	−0.0060 (12)	0.0127 (11)	−0.0152 (11)
C4	0.0565 (14)	0.0716 (17)	0.0399 (12)	−0.0003 (12)	0.0174 (11)	−0.0033 (12)
C5	0.0482 (13)	0.0532 (14)	0.0467 (12)	−0.0033 (11)	0.0100 (10)	0.0051 (11)
C6	0.0486 (13)	0.0370 (11)	0.0390 (11)	−0.0009 (9)	0.0038 (9)	0.0007 (9)
C7	0.0452 (11)	0.0308 (10)	0.0318 (10)	0.0007 (9)	0.0034 (8)	0.0015 (8)
C8	0.0354 (10)	0.0285 (10)	0.0341 (10)	0.0027 (8)	−0.0007 (8)	−0.0006 (8)
C9	0.0412 (11)	0.0314 (10)	0.0314 (10)	−0.0012 (8)	0.0024 (8)	−0.0035 (8)
C10	0.0380 (11)	0.0379 (11)	0.0402 (11)	−0.0033 (9)	0.0002 (9)	−0.0109 (9)
C11	0.0460 (13)	0.0633 (15)	0.0467 (13)	−0.0057 (11)	0.0126 (10)	−0.0157 (12)
C12	0.0669 (17)	0.0763 (18)	0.0368 (12)	−0.0104 (14)	0.0154 (12)	0.0003 (12)
C13	0.0686 (16)	0.0625 (15)	0.0398 (12)	0.0039 (13)	0.0043 (11)	0.0107 (11)
C14	0.0535 (13)	0.0460 (13)	0.0391 (11)	0.0066 (10)	0.0070 (10)	0.0025 (10)

### Geometric parameters (Å, °)

**Table:**

Cl1—C2	1.738 (2)	C5—C6	1.381 (3)
Cl2—C10	1.743 (2)	C5—H5	0.93
N1—C7	1.276 (2)	C6—H6	0.93
N1—N2	1.384 (2)	C7—H7	0.93
N2—C8	1.349 (2)	C8—C9	1.499 (3)
N2—H2	0.908 (10)	C9—C10	1.387 (3)
O1—C8	1.223 (2)	C9—C14	1.389 (3)
C1—C6	1.395 (3)	C10—C11	1.383 (3)
C1—C2	1.395 (3)	C11—C12	1.371 (3)
C1—C7	1.465 (3)	C11—H11	0.93
C2—C3	1.376 (3)	C12—C13	1.372 (3)
C3—C4	1.373 (3)	C12—H12	0.93
C3—H3	0.93	C13—C14	1.383 (3)
C4—C5	1.379 (3)	C13—H13	0.93
C4—H4	0.93	C14—H14	0.93
			
C7—N1—N2	114.69 (16)	N1—C7—H7	119.9
C8—N2—N1	119.89 (15)	C1—C7—H7	119.9
C8—N2—H2	120.6 (17)	O1—C8—N2	123.33 (17)
N1—N2—H2	119.5 (17)	O1—C8—C9	123.31 (17)
C6—C1—C2	117.19 (17)	N2—C8—C9	113.33 (16)
C6—C1—C7	121.43 (18)	C10—C9—C14	117.81 (18)
C2—C1—C7	121.39 (18)	C10—C9—C8	123.31 (17)
C3—C2—C1	121.5 (2)	C14—C9—C8	118.85 (17)
C3—C2—Cl1	118.13 (17)	C11—C10—C9	121.1 (2)
C1—C2—Cl1	120.29 (15)	C11—C10—Cl2	117.69 (17)
C4—C3—C2	119.8 (2)	C9—C10—Cl2	121.17 (15)
C4—C3—H3	120.1	C12—C11—C10	119.7 (2)
C2—C3—H3	120.1	C12—C11—H11	120.1
C3—C4—C5	120.6 (2)	C10—C11—H11	120.1
C3—C4—H4	119.7	C11—C12—C13	120.6 (2)
C5—C4—H4	119.7	C11—C12—H12	119.7
C4—C5—C6	119.3 (2)	C13—C12—H12	119.7
C4—C5—H5	120.3	C12—C13—C14	119.5 (2)
C6—C5—H5	120.3	C12—C13—H13	120.3
C5—C6—C1	121.6 (2)	C14—C13—H13	120.3
C5—C6—H6	119.2	C13—C14—C9	121.3 (2)
C1—C6—H6	119.2	C13—C14—H14	119.4
N1—C7—C1	120.20 (17)	C9—C14—H14	119.4

### Hydrogen-bond geometry (Å, °)

**Table:**

*D*—H···*A*	*D*—H	H···*A*	*D*···*A*	*D*—H···*A*
N2—H2···O1^i^	0.91 (1)	1.92 (1)	2.809 (2)	166 (2)
C7—H7···O1^i^	0.93	2.53	3.301 (2)	141
C14—H14···Cl2^i^	0.93	2.75	3.620 (2)	156

Symmetry codes: (i) −*x*+3/2, *y*−1/2, *z*.
